# Effects of various inhibitory substances and immobilization on ethanol production efficiency of a thermotolerant *Pichia kudriavzevii*

**DOI:** 10.1186/s13068-020-01729-5

**Published:** 2020-05-18

**Authors:** Ifeanyi A. Ndubuisi, Qijian Qin, Guiyan Liao, Bin Wang, Anene N. Moneke, James C. Ogbonna, Cheng Jin, Wenxia Fang

**Affiliations:** 1 National Engineering Research Center for Non-Food Biorefinery, Guangxi Academy of Sciences, Nanning, China; 2 College of Life Science and Technology, Guangxi University, Nanning, China; 3 State Key Laboratory of Non-Food Biomass and Enzyme Technology, Guangxi Academy of Sciences, Nanning, China; 4 Department of Microbiology, University of Nigeria, Nsukka, Nigeria

**Keywords:** Bioethanol production, *Pichia kudriavzevii*, Thermotolerant, Immobilization, Multi-stress tolerance, Batch fermentation

## Abstract

**Background:**

Although bioethanol production has been gaining worldwide attention as an alternative to fossil fuel, ethanol productivities and yields are still limited due to the susceptibility of fermentation microorganisms to various stress and inhibitory substances. There is therefore an unmet need to search for multi-stress-tolerant organisms to improve ethanol productivity and reduce production cost, particularly when lignocellulosic hydrolysates are used as the feedstock.

**Results:**

Here, we have characterized a previously isolated *Pichia kudriavzevii* LC375240 strain which is thermotolerant to high temperatures of 37 °C and 42 °C. More excitingly, growth and ethanol productivity of this strain exhibit strong tolerance to multiple stresses such as acetic acid, furfural, formic acid, H_2_O_2_ and high concentration of ethanol at 42 °C. In addition, simple immobilization of LC375240 on corncobs resulted to a more stable and higher efficient ethanol production for successive four cycles of repeated batch fermentation at 42 °C.

**Conclusion:**

The feature of being thermotolerant and multi-stress-tolerant is unique to *P. kudriavzevii* LC375240 and makes it a good candidate for second-generation bioethanol fermentation as well as for investigating the molecular basis underlying the robust stress tolerance. Immobilization of *P. kudriavzevii* LC375240 on corncobs is another option for cheap and high ethanol productivity.

## Background

Second-generation bioethanol has been gaining worldwide attention as an alternative to fossil fuel due to the advantages of being sustainable, renewable and environmentally friendly. However, the commercialization of bioethanol is still limited by the high cost of production. Therefore, cheap and abundant non-edible feedstock from agricultural and forestry biomass, such as lignocellulosic materials of agricultural residues, food and industrial waste, are currently being investigated for large-scale production of bioethanol [1]. The bioconversion of lignocellulosic biomass to ethanol involves three major steps: pretreatment, saccharification and fermentation. Pretreatment disrupts recalcitrant structures of lignocellulosic materials to make the cellulose and hemicellulose more accessible to saccharification enzymes, thus improving the digestibility of carbohydrate polymers into fermentable sugars [2]. In the saccharification step, cell wall degrading enzymes such as cellulases, cellobiases and β-glucosidases are used to hydrolyse pretreated lignocellulosic materials into five or six carbon sugars. Fermentation is the last step where the sugars produced from the saccharification step are converted to bioethanol by fermentation microorganisms. Simultaneous saccharification and fermentation (SSF) is usually preferred for bioethanol production based on the advantages of combining enzymatic hydrolysis and fermentation in a single fermenter, simpler operation, less cost and shorter completion time. However, the drawback is the different temperature requirement between saccharification and fermentation, as high temperature is usually required for the hydrolysis step, whereas most fermentation organisms are inhibited at high temperatures. Using thermotolerant microorganisms for ethanol production provides solution to this limitation and a great strategy for reducing the cooling and sterilization cost, as well as lower the risk of bacterial contamination during SSF.

Multiple substances, such as furfural, acetic acids and formic acids that are inhibitory to the fermentation process are released during the thermo-chemical pretreatment of the biomass. Furfural and its derivatives have been shown to reduce the organism growth rate, ATP yield and ethanol production significantly [3]. Acetic and formic acids could enter the cell in associated forms, but are dissociated inside the cell leading to a decrease in intracellular pH as well as a reduction in biomass yield and availability [4]. Oxygen radicals such as H_2_O_2_ are a result of the microbial response to stressful conditions, and are harmful to cell viability and proliferation [5]. In addition, high concentrations of the produced ethanol is also inhibitory to the fermentation microorganisms by impairing cellular wall permeability, disrupting sorting and signaling functions, as well as reducing metabolic activity, thus causing a delay in cell cycle [4]. These multi-stress conditions have made commercial ethanol production from lignocellulosic biomass more challenging. Consequently, multi-stress-tolerant organisms are necessary to increase ethanol production and yield, and to reduce the production cost.

Immobilization of fermentation organisms is a strategy to improve ethanol production by entrapping the fermentation organism in adhesible surfaces or compartments. When compared to free cells, immobilized cells have many advantages including ease of biomass separation from fermentation medium at the end of production, reducing contamination risk, enhancing cell stability and viability over several operational cycles, protection of cells from inhibitory compounds, faster production and overall cost savings. Immobilization of *Saccharomyces cerevisiae* cells for bioethanol production as well as production of other bio-products has been studied extensively [6, 7]. Immobilization by adsorption of cells on solid materials or entrapment of cells in a matrix such as calcium-alginate beads and K-carrageenan for bioethanol production has been utilized and shown to be cheap, non-toxic to the cells and easily achievable [8–10]. Lignocellulosic materials, such as loofa sponge (*Luffa cylindrica*), corncob, sugarcane bagasse and coconut bract, have advantages of cost reduction, ease of immobilization, availability, reusability, higher stability and durability when compared to entrapment beads. These materials have been widely utilized for immobilization [11–14]. *S. cerevisiae* is the most widely studied and applied microorganism for ethanol production due to its robustness and other good physiological characteristics when compared to filamentous fungi, bacteria and other yeasts [15, 16]. Despite possessing the above advantages, most *S. cerevisiae* cannot be used effectively for ethanol production using SSF method as its activity is inhibited at a temperature above 40 °C. Yeast species such as *Kluyveromyces marxianus*, few *S. cerevisiae* and *Pichia kudriavzevii* that are capable of producing ethanol between 40 and 45 °C have been reported [17–20]. *P*. *kudriavzevii* is exceptionally stress tolerant and has a growing role in bioethanol production [21] and several *P. kudriavzevii* strains have been reported to grow and produce ethanol effectively at high temperatures [19, 22–24]. However, only a few strains of *P. kudriavzevii* [25, 26] have been studied for ethanol production under multiple stress conditions.

In our previous study, we isolated a *P. kudriavzevii* LC375240 strain that could grow and produce ethanol within a temperature range of 30 °C to 42 °C, and within a pH of 3 to 8 [27]. In this study, we expand upon previous work and demonstrated that *P. kudriavzevii* LC375240 is resistant to various inhibitory substances. Simple immobilization on lignocellulosic waste such as corncobs enables this thermotolerant and multi-stress-tolerant *P. kudriavzevii* strain to be stable for repeated batch production of bioethanol.

## Results

### Thermotolerant Pichia kudriavzevii produces high amount of bioethanol

**Fig. 1:**
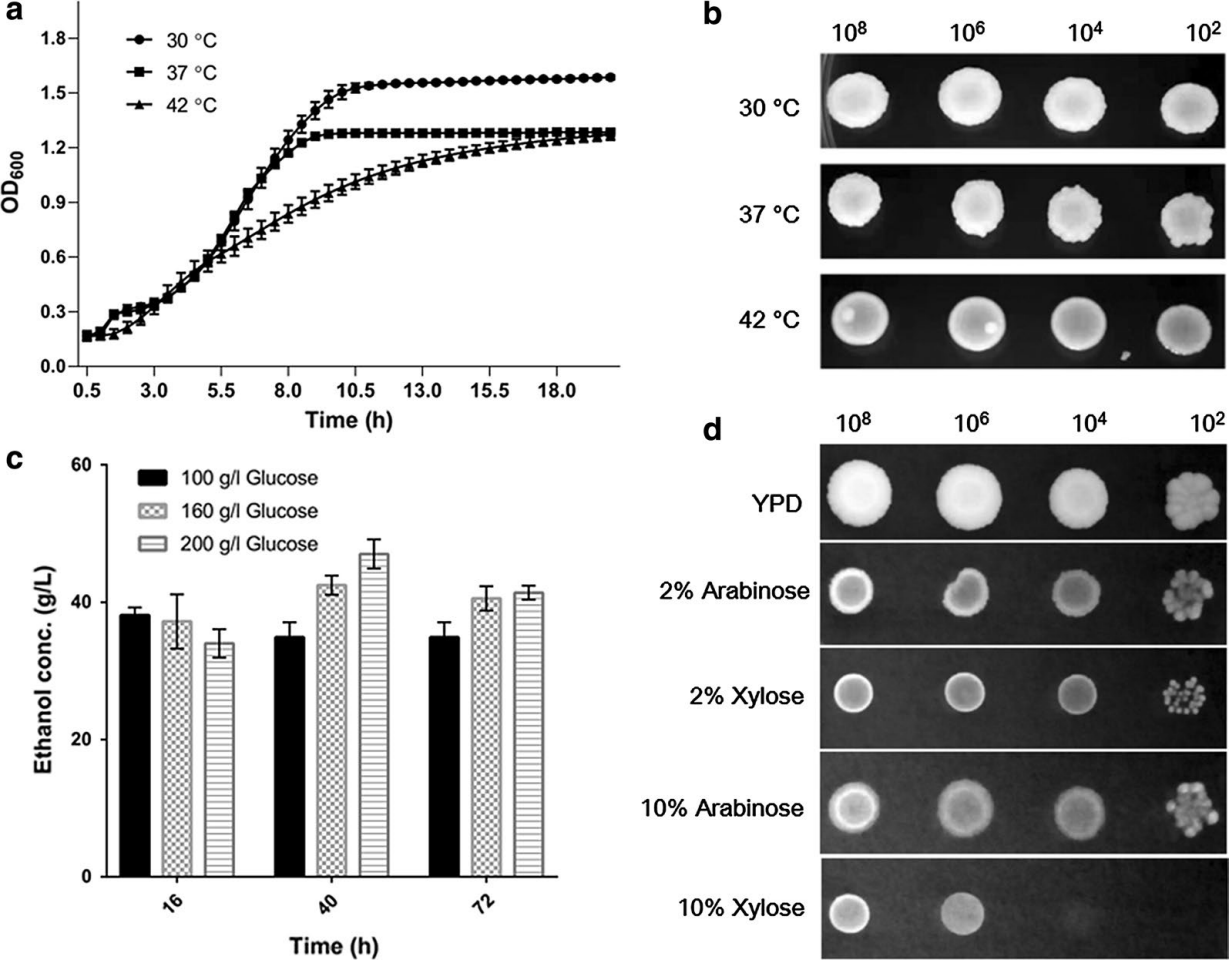
Growth of thermotolerant *P. kudriavzevii* LC375240 and ethanol production. **a** Kinetic growth curves of *P. kudriavzevii* LC375240 in YPD broth were monitored every 0.5 h interval for 20 h using plate reader for the indicated temperatures. **b** The indicated cell numbers were inoculated on YPD plates and incubated at 30 °C, 37 °C and 42 °C for 24 h. **c** Ethanol productions from YPD media with 100 g/l, 160 g/l, 200 g/l glucose were determined after 16 h, 40 h and 72 h fermentation at 42 °C. **d** The indicated cell numbers were inoculated on YPD plates or plates with two pentose sugars as sole carbon sources and incubated at 42 °C for 48 h

Although the thermotolerance feature of *P. kudriavzevii* LC375240 has been reported previously [27], the growth rate and plate spot assay have not been tested yet. Here, the growth kinetics was continuously measured in YPD broth medium at 30 °C, 37 °C and 42 °C. As shown in Fig. 1a, growth of *P. kudriavzevii* LC375240 at the initial 7.5 h was nearly the same between 30 and 37 °C, and the absorbance at 30 °C became higher and reached the stationary phase at 10 h, whereas the growth at 37 °C reached the stationary phase at 9 h with 0.14 lower OD_600_ than that at 30 °C. The growth rate at 42 °C was obviously lower before 18 h, but reached the stationary phase with nearly the same absorbance as that of 37 °C. When the spot assay on YPD plates was checked after 48 h incubation, there were no differences between the three tested temperatures even at low inoculum (Fig. 1b). It is clear that the *P. kudriavzevii* LC375240 is thermotolerant and capable of growing at 42 °C.

Bioethanol productivity is highly dependent on the concentration of the carbon sources. The ethanol production from 100 g/l glucose reached a peak value of 38.1 g/l at 16 h, and a stably retained value of 34.9 g/l at 40 h and 72 h (Table 1). The maximum ethanol concentrations of 42.5 g/l and 47.1 g/l at 40 h were obtained from glucose concentrations of 160 g/l and 200 g/l, respectively (Fig. 1c and Table 1). However, when fermentation was prolonged to 72 h the ethanol concentration produced from 160 g/l and 200 g/l glucose decreased (Fig. 1c), probably due to ethanol consumption by the yeast cells when glucose was depleted at the late stage. It is important to note that the ethanol yield decreased when the initial concentration of glucose increased (Table 1).

**Table 1:** Kinetic parameters of ethanol production with different concentrations of glucose at 42 °C

Glucose concentration (g/l)	Time (h)	Ethanol concentration (g/l)	Ethanol yields (% of the theoretical yield)	Ethanol productivity (g/l/h)
100	16	38.1 ± 1.1	74.6 ± 2.2	2.4 ± 0.1
	40	34.9 ± 2.2	68.3 ± 4.3	0.9 ± 0.1
	72	34.9 ± 2.2	68.3 ± 4.3	0.5 ± 0.0
160	16	37.2 ± 4.0	45.5 ± 4.9	2.3 ± 0.3
	40	42.5 ± 1.4	52.0 ± 1.7	1.1 ± 0.0
	72	40.6 ± 1.8	49.6 ± 2.2	0.6 ± 0.0
200	16	34.0 ± 2.1	33.2 ± 2.1	2.1 ± 0.1
	40	47.1 ± 2.1	46.0 ± 2.1	1.2 ± 0.1
	72	41.4 ± 1.0	40.5 ± 1.0	0.6 ± 0.0

Data are presented as the mean ± SD of the results from three biological replicates

When lignocellulosic hydrolysates are used as feedstock, high amount of pentose sugars such as d-xylose or l-arabinose are released from hemicellulose [28]. When we used those two pentose sugars as sole carbon sources our *P. kudriavzevii* LC375240 strain could grow, but at a slower rate than in glucose medium at both 37 °C and 42 °C (Fig. 1d). Particularly, growth on 10% xylose is severely restricted compared to 10% arabinose plates. Moreover, nearly no ethanol was produced from the pentose media due to the limited growth, suggesting that our *P. kudriavzevii* strain could assimilate pentose sugars for growth, but could not ferment them to produce ethanol.

### Stress tolerance of *P. kudriavzevii* LC375240 to furfural and formic acid

**Fig. 2:**
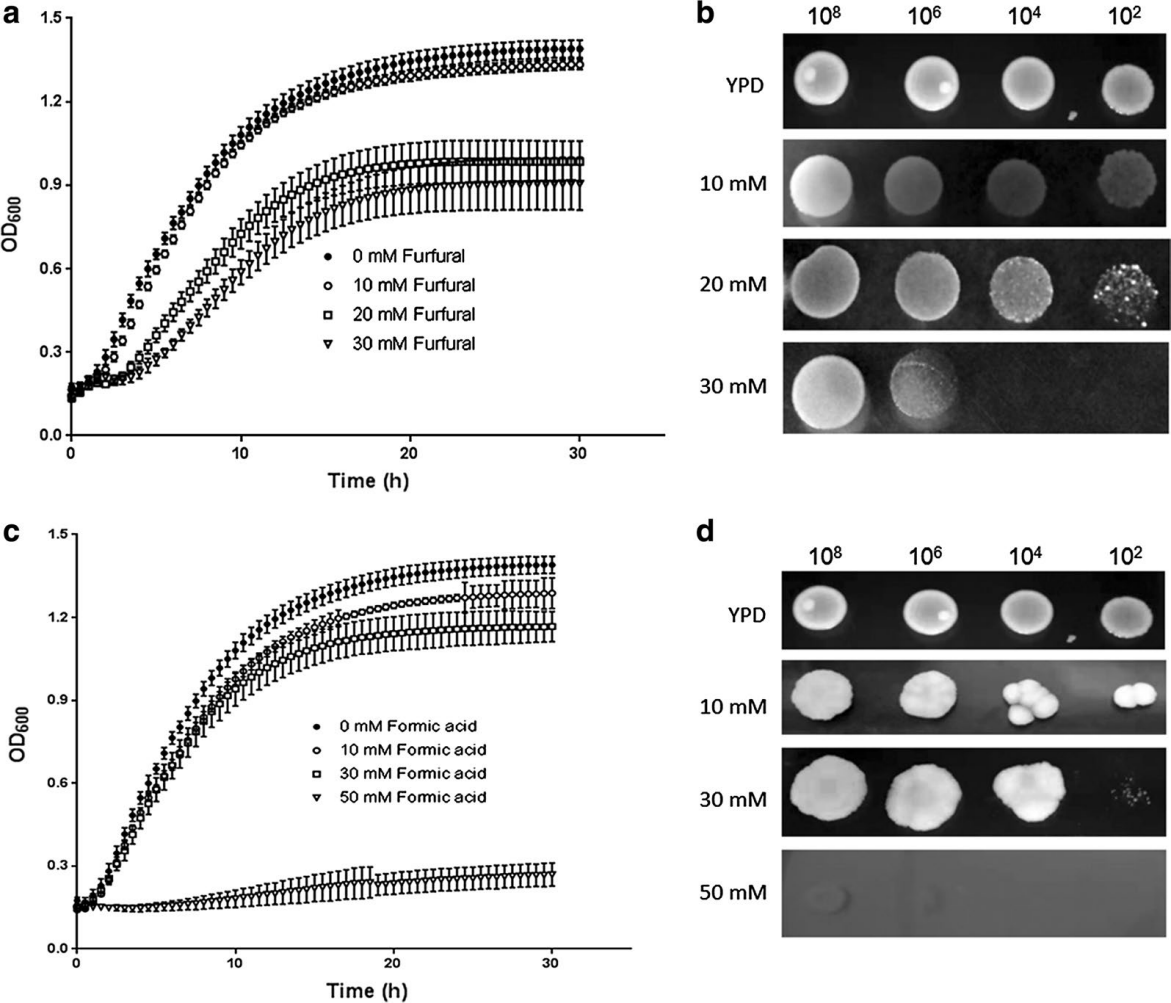
Growth of *P. kudriavzevii* LC375240 in the presence of furfural and formic acid. **a** Kinetic growth curves of LC375240 in YPD broth supplemented with various concentrations of furfural were monitored using plate reader every 0.5 h interval for 30 h at 42 °C. **b** The indicated cell numbers were inoculated on YPD plates supplemented with various concentrations of furfural and incubated at 42 °C for 48 h. **c** Kinetic growth curves of LC375240 in YPD broth supplemented with various concentrations of formic acid were monitored using plate reader every 0.5 h interval for 30 h at 42 °C. **d** The indicated cell numbers were inoculated on YPD plates supplemented with various concentrations of formic acid and incubated at 42 °C for 48 h

During the pretreatment and saccharification steps of lignocellulosic materials many by-products such as acetic acid, furfural and formic acid are released. These compounds inhibit the growth of fermentation microorganisms and thus reduce the bioethanol production. In our previous study, the LC375240 strain exhibited high tolerance to 70 mM acetic acid [27], implying it is a stress-tolerant strain. In order to know whether it is resistant to the other two agents, we tested its growth in the presence of furfural and formic acid at 42 °C. As shown in Fig. 2a, the growth of LC375240 in the presence of 10 mM furfural was nearly the same as in YPD medium. However, the growth slightly decreased in the presence of 20 mM and 30 mM furfural. Consistently, the same inhibition trend was seen on YPD plates with furfural supplementation (Fig. 2b).

The effect of formic acid on the growth of LC375240 was also tested. Interestingly, the LC375240 grew well in the presence of 10 mM and 30 mM formic acid both in liquid cultivation and on YPD plates (Fig. 2c, d). However, in a medium with 50 mM formic acid the growth of LC375240 was severely repressed. Taken together, *P. kudriavzevii* LC375240 exhibits stress tolerance against furfural and formic acid.

### Ethanol production by *P. kudriavzevii* LC375240 in the presence of furfural, acetic acid and formic acid

The effects of furfural, acetic acid and formic acid on ethanol production were evaluated at 42 °C fermentation, and kinetic parameters that were calculated are listed in Table 2. In the presence of 10 mM and 20 mM furfural relatively high ethanol concentration, ethanol yield and ethanol productivity were obtained, although ethanol production was significantly reduced in 30 mM furfural (Table 2). Our *P. kudriavzevii* LC375240 strain maintained high ethanol production even in the presence of 70 mM acetic acid with yield of 63% at 40 h and 72 h (Table 2). Similarly, even in the presence of 45 mM formic acid the ethanol concentration reached 32 g/l at 72 h, although the production was relatively lower at 16 h and 40 h compared to the amount in 25 mM and 35 mM formic acid. However, when mixed combinations such as 10 mM furfural with 40 mM acetic acid or 10 mM furfural with 25 mM formic acid were added, the LC372540 strain could not grow well at 42 °C, therefore the ethanol production was too low to be detected. Nevertheless, our *P. kudriavzevii* LC375240 strain exhibited sound ethanol production at 42 °C in the presence of furfural, acetic acid and formic acid.

**Table 2:** Kinetic parameters of ethanol production from 100 g/l glucose in the presence of inhibitory substances at 42 °C

Inhibitors [29]	Time (h)	Ethanol concentration (g/l)	Ethanol yields (% of the theoretical yield)	Ethanol productivity (g/l/h)
Furfural				
10	16	29.0 ± 1.6	56.8 ± 3.1	1.8 ± 0.1
	40	37.5 ± 4.3	73.4 ± 8.4	0.9 ± 0.1
	72	36.3 ± 3.8	71.0 ± 7.4	0.5 ± 0.1
20	16	29.0 ± 1.2	56.8 ± 2.3	1.8 ± 0.1
	40	35.6 ± 0.8	69.7 ± 1.6	0.9 ± 0.0
	72	31.7 ± 1.3	62.0 ± 2.5	0.4 ± 0.0
30	16	17.8 ± 2.0	34.8 ± 3.9	1.1 ± 0.1
	40	26.8 ± 2.9	52.4 ± 5.7	0.7 ± 0.1
	72	28.1 ± 1.0	55.0 ± 2.0	0.4 ± 0.0
Acetic acid				
40	16	28.4 ± 0.9	55.6 ± 1.8	1.8 ± 0.1
	40	41.2 ± 0.8	80.6 ± 1.6	1.0 ± 0.0
	72	37.8 ± 4.7	74.0 ± 9.2	0.5 ± 0.1
50	16	27.3 ± 1.7	53.4 ± 3.3	1.7 ± 0.1
	40	35.3 ± 3.1	69.1 ± 6.1	0.9 ± 0.1
	72	39.9 ± 1.1	78.1 ± 2.2	0.6 ± 0.0
70	16	23.1 ± 2.4	45.2 ± 4.7	1.4 ± 0.2
	40	32.4 ± 1.7	63.4 ± 3.3	0.8 ± 0.0
	72	32.6 ± 1.7	63.8 ± 3.3	0.5 ± 0.0
Formic acid				
25	16	36.1 ± 1.2	70.6 ± 2.3	2.3 ± 0.1
	40	33.4 ± 2.5	65.4 ± 4.9	0.8 ± 0.1
	72	32.8 ± 3.9	64.2 ± 7.6	0.5 ± 0.1
35	16	28.8 ± 1.5	56.4 ± 2.9	1.8 ± 0.1
	40	34.2 ± 1.0	66.9 ± 2.0	0.9 ± 0.0
	72	32.6 ± 2.6	63.8 ± 5.1	0.5 ± 0.0
45	16	20.3 ± 1.1	39.7 ± 2.2	1.3 ± 0.1
	40	27.7 ± 3.5	54.2 ± 6.8	0.7 ± 0.1
	72	32.0 ± 1.8	62.6 ± 3.5	0.4 ± 0.0

Data are presented as the mean ± SD of the results from three biological replicates

### Tolerance of *P. kudriavzevii* LC375240 to hydrogen peroxide and ethanol

**Fig. 3:**
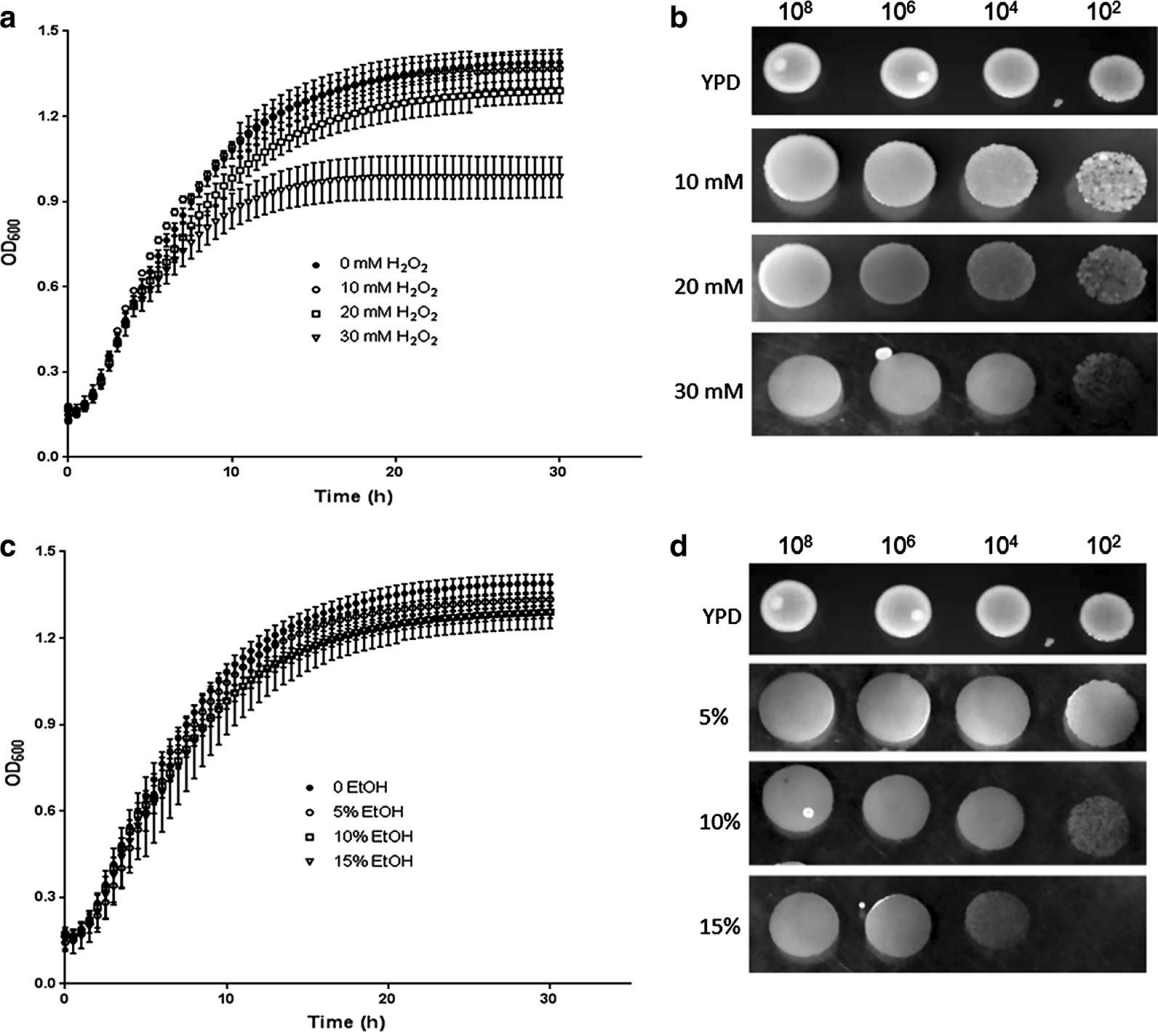
Tolerance of *P. kudriavzevii* LC375240 to H_2_O_2_ and ethanol. **a** Kinetic growth curves of LC375240 in YPD broth supplemented with various concentrations of H_2_O_2_ were monitored using plate reader every 0.5 h interval for 30 h at 42 °C. **b** The indicated cell numbers were inoculated on YPD plates supplemented with various concentrations of H_2_O_2_ and incubated at 42 °C for 48 h. **c** Kinetic growth curves of LC375240 in YPD broth supplemented with various percentages of ethanol were monitored using plate reader every 0.5 h interval for 30 h at 42 °C. **d** The indicated cell numbers were inoculated on YPD plates supplemented with various percentages of ethanol and incubated at 42 °C for 48 h

During industrial fermentation, oxidative stress is commonly induced as well as increased accumulation of bioethanol. Therefore we investigated the effects of hydrogen peroxide (H_2_O_2_) and ethanol on the growth of *P. kudriavzevii* LC375240 at 42 °C. As shown in Fig. 3a, the strain grew well in the presence of 10 mM and 20 mM H_2_O_2_ in liquid culture, although the growth slightly dropped in the presence of 30 mM H_2_O_2_, demonstrating that LC375240 is tolerant to H_2_O_2_. This is also evident from the data in Fig. 3b as the strain grew well on YPD plates supplemented with H_2_O_2_ up to 30 mM.

Although ethanol is the target product of bioethanol fermentation, it is also an inhibitory factor to the fermentation organisms particularly as the amount increases. However, our LC375240 strain displayed remarkable tolerance towards ethanol even at a concentration of 15% in both liquid culture (Fig. 3c) and on YPD plates (Fig. 3d). Obviously, strain LC375240 is highly tolerant to H_2_O_2_ and ethanol.

### Immobilization of *P. kudriavzevii* LC375240 on supporting materials

**Fig. 4:**
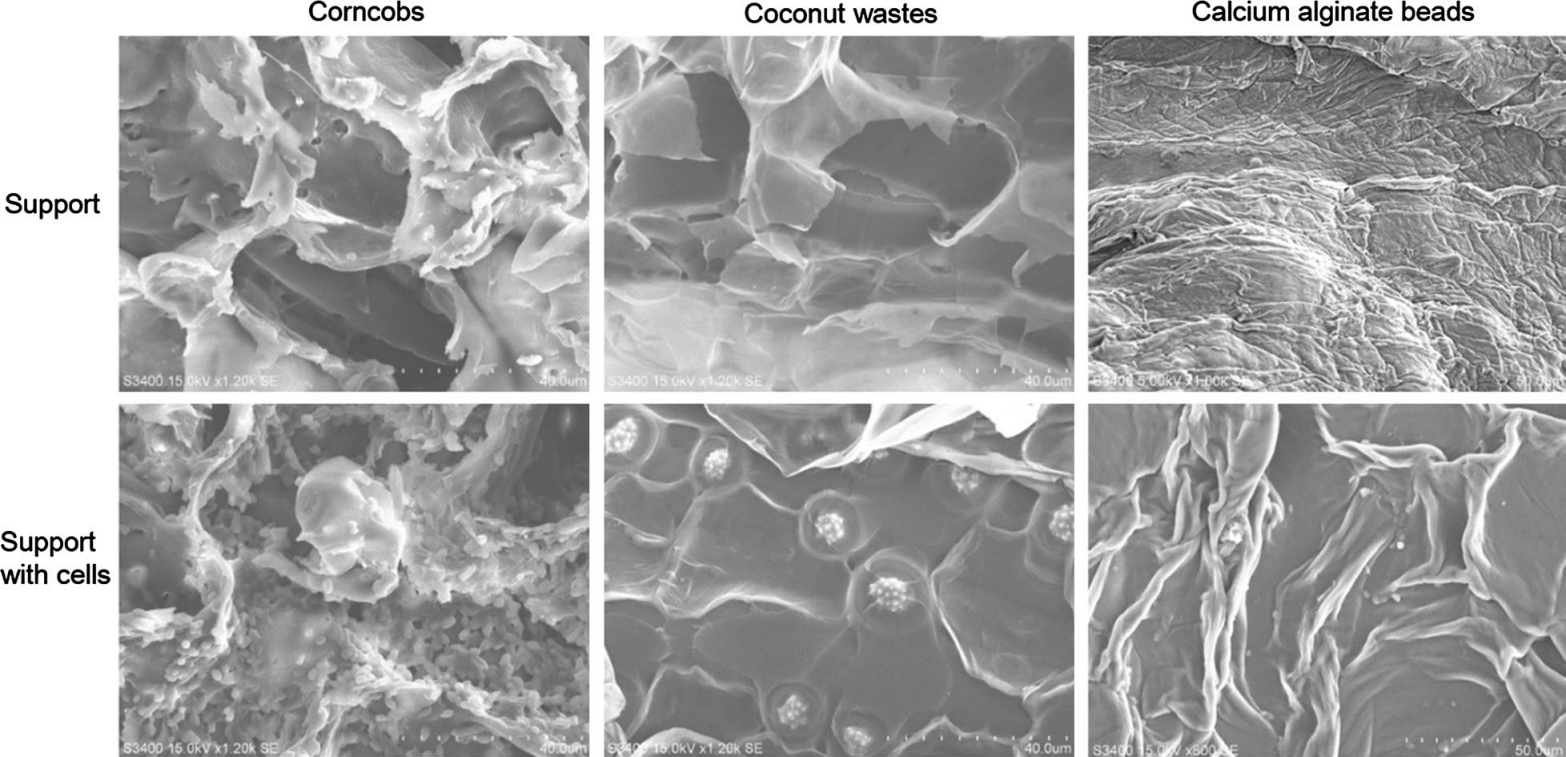
Immobilization of *P. kudriavzevii* LC375240. Pieces of corncobs and coconut wastes or calcium alginate beads with entrapped cells were added into YPD medium for yeast cells cultivation at 30 °C for 24 h with 180 rpm shaking. The immobilization images from the supports were captured using scanning electron microscope. Immobilized beads were cut to slices before being captured. Scale bar is represented as dotted line in each image

Immobilization of yeast cells for ethanol production is a strategy used to protect cells from inhibitory compounds and stress conditions, to increase fermentation efficacy and to save cost in repeated batch fermentations. Calcium alginate beads are usually used to entrap the yeast cells. Here, we conducted immobilization of LC375240 not only in calcium alginate beads, but also on corncobs and coconut wastes, and we confirmed their immobilization efficacy using scanning electron microscopy (SEM). Surprisingly as shown by SEM in Fig. 4, very few *P. kudriavzevii* LC375240 cells were entrapped in the beads, relatively few cells were fixed on coconut wastes with clump and shrink morphology, whereas large amount of cells were efficiently attached to corncobs with well-stretched and separate morphology. It is reasonable to conclude that corncobs are the best supporting material for the immobilization of LC375240 cells when compared to coconut wastes and commonly used calcium alginate beads.

### Repeated batch fermentation using free cells and immobilized *P. kudriavzevii* LC375240

To evaluate the effectiveness of the immobilized cells for ethanol production, repeated batch fermentations were conducted and compared to free cells. As shown in Table 3, cells immobilized on both corncobs and coconut wastes displayed 1.1 g/l/h of ethanol productivity which was significantly higher than the values obtained with free cells in the first and second batches of fermentation (*p* values are listed in Table 3). There was no significant statistical difference in ethanol productivity between corncobs immobilized cells and the free cells at the third and fourth batches of fermentation. Whereas compared to free cells, immobilized cells on coconut wastes showed no difference in third batch, there was a significantly reduced ethanol production for coconut waste, while corncobs and free cells remain stable at the fourth batch (Table 3). No statistical difference in ethanol production was observed between cells immobilized in calcium alginate beads and the free cells for the first, second and third batches, but significantly reduced ethanol production of beads-immobilized cells in the fourth batch of fermentation, which is probably due to bursting of the beads (Table 3). On the whole, free cells of *P. kudriavzevii* LC375240 were suitable for repeated batch fermentation, but immobilization on corncobs rendered more stable and higher efficient ethanol production throughout the repeated batch fermentation.

**Table 3:** Summary of bioethanol production from immobilized cells and free cells in repeated batch fermentation with 100 g/l glucose at 42 °C for 40 h per batch

Immobilization	Batch	Ethanol conc. (g/l)	Ethanol yields (% of the theoretical yield)	Ethanol productivity (g/l/h)	Statistical *p* values
Free cells	1	36.9 ± 2.4	72.2 ± 4.7	0.9 ± 0.1	–
	2	40.6 ± 0.8	79.5 ± 1.6	1.0 ± 0.0	–
	3	42.1 ± 1.7	82.5 ± 3.3	1.1 ± 0.0	–
	4	42.5 ± 1.4	83.1 ± 2.7	1.1 ± 0.0	–
Corncobs	1	42.1 ± 0.7	82.4 ± 1.4	1.1 ± 0.0	0.0004***
	2	42.3 ± 1.1	82.7 ± 2.2	1.1 ± 0.0	0.01*
	3	42.6 ± 2.2	83.4 ± 4.3	1.1 ± 0.1	0.7, ns
	4	44.5 ± 1.9	87.1 ± 3.7	1.1 ± 0.0	0.06, ns
Coconut wastes	1	44.9 ± 0.5	87.9 ± 1.0	1.1 ± 0.0	0.00001***
	2	44.4 ± 2.2	86.9 ± 4.3	1.1 ± 0.1	0.003**
	3	41.4 ± 2.2	81.0 ± 4.3	1.0 ± 0.1	0.5, ns
	4	36.0 ± 2.6	70.5 ± 5.1	0.9 ± 0.1	0.0004***
Calcium alginate beads	1	38.3 ± 1.2	74.9 ± 2.3	1.0 ± 0.0	0.2, ns
	2	39.4 ± 1.4	77.0 ± 2.7	1.0 ± 0.0	0.08, ns
	3	42.2 ± 2.0	82.6 ± 3.9	1.1 ± 0.1	1.0, ns
	4	34.9 ± 1.7	68.3 ± 3.3	0.9 ± 0.0	0.000008***

Data are presented as the mean ± SD of the results from three biological replicates. One-way analysis of variance (ANOVA) was used to compare the ethanol production between the same batch of immobilized groups and the free cells group. Not significant *p *> 0.05, ns; statistically significant *p *≤ 0.05*; very significant *p *≤ 0.01**; highly significant *p *≤ 0.001***

## Discussion

Despite the increased worldwide attention on bioethanol production, the microorganisms for fermentation still encounter multiple challenges, particularly when using lignocellulosic hydrolysates as the feedstock during which multiple inhibitory substances are usually released. On top of these, thermotolerant yeast strains are required for SSF to reduce cost and avoid contamination. Several strategies such as over-liming of acid-treated biomass, adaptation of the fermentation microorganisms in the presence of toxic compounds and genetic engineering of microorganisms to improve resistance to inhibitory factors have been performed [3, 30]. However, these methods are time consuming and not very efficient. In a previous study, we isolated a thermotolerant *P. kudriavzevii* LC375240 from spoilt fruit, which exhibited tolerance to acetic acid up to 70 mM [27]. Here, we confirmed the thermotolerant feature of this strain by studying its growth kinetics in liquid medium as well as growth on solid plates (Fig. 1a, b). The present study has shown that high glucose concentrations reduced ethanol yields at 42 °C, probably due to the limited efficiency of converting glucose to ethanol (Fig. 1c). Interestingly, *P. kudriavzevii* LC375240 exhibited appreciable growth and ethanol production as well as tolerance to furfural, formic acid, H_2_O_2_ and ethanol at 42 °C (Figs. 2, 3 and Table 2). Several publications have reported that some *P. kudriavzevii* strains possess multi-stress tolerance [19, 30, 31]. These include *P. kudriavzevii* N77-4 which could tolerate 100 mM acetic acid, but was sensitive to 6% ethanol at 30 °C [31]; *P. kudriavzevii* RZ8-1 that could grow on medium containing 5 g/l acetic acid (approximately 82.5 mM) or higher than 12% ethanol at 35 °C [19], and soil-isolated *P. kudriavzevii that* could grow at 12% ethanol, 1.44 g/l (approximately 15 mM) furfural and 2.5 g/l (approximately 41 mM) acetic acid [32]. Compared to these reported strains, *P. kudriavzevii* LC375240 clearly showed the highest tolerance to the inhibitory substances at 42 °C, demonstrating that *P. kudriavzevii* LC375240 is a better thermotolerant and multi-stress-tolerant strain for bioethanol production.

Heat-shock proteins usually account for thermotolerance, whereas alcohol dehydrogenase is associated with ethanol production. In a previous study, it was reported that genes encoding heat-shock proteins such as *ssq1* and *hsp90*, and alcohol dehydrogenases such as *adh1*, *adh2*, *adh3*, *adh4* as well as glyceraldehyde-3-phosphate dehydrogenase *tdh2* were up-regulated in a thermotolerant ethanol-producing *P. kudriavzevii* [19]. Genes such as *ydeP*, *yhiE* or *ydeO* were validated as acid resistance genes in *E. coli* since their deletion resulted in the elimination of resistance properties, whereas their overexpression conferred resistance to exponentially growing cells [33]. In addition, increased expression of either *RCN1* or *RSA3* genes improved the tolerance of wine yeast strains to ethanol, heat, osmotic and oxidative stresses [34]. It is therefore, necessary to investigate the expression level of those reported genes in our thermotolerant and multi-stress-tolerant *P. kudriavzevii* LC375240 strain in the future. Moreover, combination of genomic sequencing and transcriptomic analysis will probably reveal the novel genes that render multi-stress-tolerant feature to LC375240.

Utilization of pentose to produce bioethanol is important for the efficient conversion of lignocellulose biomass since some cellulosic hydrolysates consist of nearly 30–40% pentose such as xylose [35]. Our *P. kudriavzevii* LC375240 strain could use xylose and arabinose for growth, but at a slower rate than using glucose, and this is indicating the slower utilization efficiency of pentose (Fig. 1d). Higher concentration of xylose repressed growth, implying substrate inhibition towards xylose metabolic pathway. Genetic engineering of efficient pentose transport system, driving pentose to pentose phosphate pathway or natural selection of efficient pentose utilization strain are suggested approaches to improve the pentose utilization of LC375240 for ethanol production [35].

Immobilization of yeast cells on matrix such as Ca-alginate beads offers the advantages of easy operation, reduced cost and protecting the cells from multiple stress conditions [8]. However, due to the low mass transfer efficiency and low stability, the beads-immobilized cells cannot be used over several batches. Instead immobilization of cells on lignocellulosic materials is easy, stable, reusable and cheap. Here, we compared immobilization of *P. kudriavzevii* LC375240 in Ca-alginate beads and lignocellulosic wastes (corncobs and coconut wastes). The immobilization efficiency and morphology of *P. kudriavzevii* cells on the three supporting materials were different. Immobilization on corncobs was the most efficient in terms of immobilized cell concentration and normal cell shape, whereas the alginate beads took in the least cells with shrink size (Fig. 4). Moreover, corncob-immobilized cells displayed consistent 1.1 g/l/h ethanol productivity during the four repeated batch fermentations. The stability and high ethanol production was better than free cells and cells immobilized by beads or coconut wastes.

Although there are not many reports on the immobilization of *P. kudriavzevii* for direct comparison, Zichova´ et al. reported that ethanol production by poly(vinyl alcohol) hydrogel-entrapped *P. kudriavzevii* was higher than that produced by the free cells at 40 °C [14]. They also reported that the free cells of *P. kudriavzevii* lost viability after 90 h of fermentation at 40 °C. In contrast, high ethanol production of *P. kudriavzevii* LC375240 was observed after four cycles of fermentation at 42 °C in this study. Laopaiboon and Laopaiboon (2012) reported that corncob-immobilized *S. cerevisiae* TISTR 5048 gave significantly higher ethanol concentration, yield and productivity than those immobilized on calcium alginate beads in repeated batch ethanol production [36]. Singh et al. reported that ethanol production from sugarcane bagasse-immobilized *S. cerevisiae* was higher than cells immobilized on Ca-alginate matrices [6]. The work also reported that *S. cerevisiae* immobilized on sugarcane bagasse could last up to 10 cycles in a repeated batch fermentation while the same cells immobilized in Ca-alginate matrix lasted only 4 cycles. Ethanol production by *Candida shehatae* ATCC 22984 immobilized on terracotta beads, coconut bract and corncobs gave an average ethanol concentration of 17.03 g/l, 17.20 g/l and 16.40 g/l, respectively, after 5 cycles of fermentation while free cells had an average of 16.78 g/l under the same condition [37]. These reports are in line with our finding that ethanol production is improved by immobilization, particularly on lignocellulosic materials such as corncobs with higher stability, durability and reusability when compared to entrapment in Ca-alginate matrix.

## Conclusion

Being a thermotolerant strain for bioethanol production *P. kudriavzevii* LC375240 grew well at 42 °C at which conversional yeasts usually cannot survive. It produced high amount of ethanol from glucose, but could not utilize pentose such as xylose and arabinose to produce ethanol. It also exhibits strong tolerance to stresses such as acetic acid, furfural, formic acid, H_2_O_2_ and ethanol at 42 °C. This multi-stress tolerance feature is unique to *P. kudriavzevii* LC375240 and makes it a good candidate strain for industrial application. Investigating the underlying tolerance mechanisms and using genetic manipulations to transfer the stress tolerance feature or genes to existing industrial strains are of particular interest for building stress-tolerant cell factories. In addition, simple immobilization of *P. kudriavzevii* LC375240 on corncobs led to stable production of ethanol in repeated batch fermentation, indicating that immobilization is another cheap option for high ethanol productivity.

## Materials and method

### Strain

*Pichia kudriavzevii* LC375240 used in this study was isolated from a spoilt fruit in Nigeria. The isolation, screening and identification procedure have been reported in a previous publication [27].

### Growth characterization of *P. kudriavzevii* LC375240 under different stress conditions

Growth of *P. kudriavzevii* LC375240 under different stress conditions (temperature, ethanol, formic acid, furfural and H_2_O_2_) was determined in both liquid medium and on solid agar plates. A single yeast colony was inoculated into YPD medium (10 g/l yeast extract, 20 g/l peptone, 20 g/l glucose) and incubated at 37 °C on a rotary incubator shaker at 200 rpm. After overnight incubation, the cells were harvested by centrifugation at 12,000 rpm for 3 min, and then washed with distilled water. They were diluted to optical density at 600 nm (OD_600_) of 0.1 in a 96-well plate and read by INFINITE 200PRO plate reader every 0.5 h at the setting conditions. For temperature stress, the plate was incubated at 30 °C, 37 °C or 42 °C for 20 h. For other stress conditions, the YPD medium was supplemented with different concentrations of the inhibitory compounds; furfural (10 mM, 20 mM and 30 mM), formic acid (10 mM, 30 mM and 50 mM), ethanol (5%, 10% and 15%) and H_2_O_2_ (10 mM, 20 mM and 30 mM), and incubated at 42 °C. The optical densities were measured every 0.5 h at 600 nm over a period of 30 h or longer. The sensitivity of the cells to these stresses during cultivation on agar plates was also investigated. In this case, overnight culture was centrifuged, washed with distilled water and then reconstituted with sterile distilled water. The cells were counted using haemocytometer. It was serially diluted from 2 × 10^8^ cells/ml to 2 × 10^2^ cells/ml and 5-µl aliquots of 10^8^, 10^6^, 10^4^ and 10^2^ dilutions were spotted on YPD agar plates supplemented with various concentrations of the above inhibitors. To test growth on pentose sugars, xylose and arabinose were used to replace glucose in YPD to make plates, and the above inoculum was applied. The plates were incubated at 42 °C for 48 h and then used for photo-imaging.

### Effect of carbon source concentration on ethanol production

The effect of carbon source concentration on ethanol production was investigated in YPD liquid medium with 100 g/l, 160 g/l and 200 g/l glucose. Fermentations were performed at 42 °C on a rotary shaker at 200 rpm. Samples were withdrawn at 16 h, 40 h and 72 h to determine the ethanol concentration.

### Immobilization of *P. kudriavzevii* LC375240

Three immobilization support materials (corncobs, coconut wastes and calcium alginate beads) were used for immobilization. For immobilization in calcium alginate beads 2% sodium alginate and 2% calcium chloride dihydrate were autoclaved at 121 °C for 15 min and allowed to cool. 2 × 10^8^ of the yeast cells were mixed with 20 ml of the sodium alginate solution. The sodium alginate solution containing the yeast cells was gradually dropped into 80 ml of ice-cold CaCl_2_ solution using a dropper. The beads were allowed to harden at 4 °C for 24 h. Then the beads were washed with sterile water and stored in sterile water at 4 °C. A total of about 40 beads were produced and each bead contained about 5 × 10^6^ cells.

For immobilization on other support materials the corncobs and coconut wastes were cut to a size of approximately 1 cm × 2 cm. They were washed and dried overnight at 70 °C. The dried supports were autoclaved at 121 °C for 20 min and allowed to cool. 15 pieces of each support were added to 70 ml of YPD medium in a 250-ml conical flask. The flasks were inoculated with 0.5 ml of yeast cell suspension containing a total of 2 × 10^8^ cells and incubated at 30 °C and 180 rpm for 24 h before use.

The images of the support materials with or without yeast cells were captured using scanning electron microscope. For the beads, they were cut before capturing the images.

### Repeated batch fermentation using immobilized and free yeast cells

Normalization of the inoculum of the fermentation was based on the initial cell concentration before incubation. For cells immobilized in calcium alginate beads, 15 beads were added per flask giving an initial cell concentration of 7.5 × 10^7^ per flask. In the case of the cells immobilized on corncobs and coconut fibers, three pieces of the fiber/corncobs were used. If all the cells got attached the initial cell concentration was 4 × 10^7^ cells per flask. However, the immobilization efficiency was not 100% and during the 24 h of immobilization procedure the attached cells also grew, so the actual cell concentration was more than 4 × 10^7^ cells per flask. Free cells of 7.5 × 10^7^ and the above inoculum from the support materials were inoculated into the 250-ml Erlenmeyer flask containing 100 ml of 10% YPD. Cultures were incubated in a shaking incubator at 42 °C and 200 rpm for 40 h per cycle. At the end of each fermentation run, the supports were collected from fermentation broth and washed with sterile distilled water then transferred to fresh media for subsequent batch fermentation. Free cells after each batch fermentation were centrifuged, 90% of the supernatant was discarded and replaced with fresh broth. Samples were taken at the end of each batch and analyzed for ethanol concentration.

### Ethanol determination and data analysis

Ethanol concentration was analyzed by a gas chromatograph (GC-7890A, Agilent Technologies, USA) with a flame ionization detector using a glass column packed with FID (N_2_ gas flow rate = 30 ml/min, H_2_ = 30 ml/min, air = 300 ml/min, injector 150 °C, column 100 °C, detector 250 °C). Calculation of ethanol concentration was based on the equation showing linear relationship between ethanol peak area and acetonitrile peak area. The theoretical ethanol yields using 100 g/l, 160 g/l and 200 g/l glucose are 51.1 g/l, 81.8 g/l and 102.3 g/l, respectively. The ethanol yield was calculated as the percentage ratio of actual ethanol yield to the theoretical ethanol yield. Ethanol productivity was calculated as the ratio of ethanol concentration (g/l) to the fermentation time (h). All fermentation experiments were performed in triplicates and all the experimental numerical data were expressed as the mean ± SD. One-way ANOVA was used for statistical analysis, where *p *> 0.05 showing not significant; *p *≤ 0.05 showing statistically significant; *p *≤ 0.01 showing very significant and *p *≤ 0.001 showing highly significant. The final figure was made by GraphPad Prism 7.0.

## Data Availability

All data generated or analyzed during this study are included in this article.

## Abbreviations

SSF: Simultaneous saccharification and fermentation
H_2_O_2_: Hydrogen peroxide
SEM: Scanning electron microscopy
